# Prognostic role of *METTL1* in glioma

**DOI:** 10.1186/s12935-021-02346-4

**Published:** 2021-11-27

**Authors:** Lun Li, Yi Yang, Zhenshuang Wang, Chengran Xu, Jinhai Huang, Guangyu Li

**Affiliations:** 1 Department of Neurosurgery, Anshan Hospital of the First Hospital of China Medical University, Anshan, China; 2 Department of Neurosurgery, First Affiliated Hospital of China Medical University, Shenyang, China; 3 Department of Orthopedics, First Affiliated Hospital of China Medical University, Shenyang, China

**Keywords:** Methyltransferase-like 1, Glioma, siRNA, CCK8, Biomarker, Nomogram, Prognosis

## Abstract

**Background:**

Current treatment options for glioma are limited, and the prognosis of patients with glioma is poor as the available drugs show low therapeutic efficacy. Furthermore, the molecular mechanisms associated with glioma remain poorly understood. *METTL1* mainly catalyzes the formation of N(7)-methylguanine at position 46 of the transfer RNA sequence, thereby regulating the translation process. However, the role of *METTL1* in glioma has not been studied to date. The purpose of this study was to analyze the expression and prognosis of METTL1 in glioma, and to explore the potential analysis mechanism.

**Methods:**

Data from five publicly available databases were used to analyze *METTL1* expression across different tumor types and its differential expression between carcinoma and adjacent normal tissues. The expression of *METTL1* in glioma was further validated using real-time polymerase chain reaction and immunohistochemistry. Meanwhile, siRNA was used to knockdown *METTL1* in U87 glioma cells, and the resultant effect on glioma proliferation was verified using the Cell Counting Kit 8 (CCK8) assay. Furthermore, a nomogram was constructed to predict the association between *METTL1* expression and the survival rate of patients with glioma.

**Results:**

*METTL1* expression increased with increasing glioma grades and was significantly higher in glioma than in adjacent noncancerous tissues. In addition, high expression of *METTL1* promoted cell proliferation. Moreover, *METTL1* expression was associated with common clinical risk factors and was significantly associated with the prognosis and survival of patients with glioma. Univariate and multivariate Cox regression analyses revealed that *METTL1* expression may be used as an independent prognostic risk factor for glioma. Furthermore, results of functional enrichment and pathway analyses indicate that the mechanism of *METTL1* in glioma is potentially related to the MAPK signaling pathway.

**Conclusions:**

High *METTL1* expression is significantly associated with poor prognosis of patients with glioma and may represent a valuable independent risk factor. In addition, high expression of *METTL1* promotes glioma proliferation and may regulate mitogen-activated protein kinase (MAPK) signaling pathway. Thus, *METTL1* may be a potential biomarker for glioma. Further investigations are warranted to explore its clinical use.

**Supplementary Information:**

The online version contains supplementary material available at 10.1186/s12935-021-02346-4.

## Background

Glioma derived from glial cells is the most common malignant tumor of the central nervous system and accounts for more than half of all malignant brain tumors [1, 2]. The clinical features of malignant glioma include poor prognosis, high recurrence rate, and high mortality [3]. Currently, gliomas are primarily treated by surgical resection, adjuvant postoperative radiotherapy, and chemotherapy, and targeted therapy; however, the treatment efficacy has not been satisfactory [4]. Hence, there is an urgent need to explore the molecular mechanisms associated with glioma and identify effective therapeutic targets.

The methyltransferase-like 1 (*METTL1*) gene, located on human chromosome 12, is responsible for mediating the formation of N(7)-methylguanine (m^7^G) and regulating mRNA translation [5]. Recently, upregulation of *METTL1* expression was shown to promote oncogenic activity and modulate resistance to antitumor drugs. In a study, overexpression of *METTL1* enhanced the cytotoxic effect of cisplatin on cisplatin-resistant colon cancer cells by mir149-3p action via the S100A4/p53 signaling pathway [6], and induced the expression of NANOG and Kruppel Like Factor 4 (KLF4) [7], two phosphatase and tensin homolog (PTEN-regulated molecules [8], and promoted carcinogenicity in hepatocellular carcinoma (HCC) via the PTEN/AKT axis [9]. *METTL1* together with WDR4 promotes the methylation of transfer and ribosomal RNAs [10], thereby regulating mRNA translation [11]. At the same time, it also affects miRNA functions [12], gene regulation, and splicing [13]. *METTL1* is expressed in various tissues and organs and performs several functions. For example, *METTL1*, as a regulator of embryonic stem cell self-renewal and differentiation [14], affects the differentiation and function of endodermal cells in vitro [15]. Furthermore, the knockout of *METTL1* significantly promotes the differentiation and function of endothelial cells in vitro [16] and impairs the self-renewal of mouse embryonic stem cells and differentiation of nerve cells [11]. Moreover, *METTL1* is believed to be an important contributor to tumorigenesis as it regulates the sensitivity of cancer cells to the antitumor drugs cisplatin and 5-fluorouracil [17, 18]. In addition, *METTL3*, which also belongs to the gene family of *METTL1*, was found to promote cancer progression in gliomas [19, 20]. Therefore, we speculated that in addition to its role in tumors, *METTL1* expression may be increased in gliomas and may promote the progression of gliomas, thereby affecting the prognosis of patients.

In this study, the differences in the expression of METTL1 between carcinoma and paracancer cells of glioma were analyzed by bioinformatics, PCR, and immunohistochemistry experiments. *METTL1* was knocked down in vitro for functional and pathway analysis. In addition, we assessed whether *METTL1* is a potential independent prognostic risk factor in gliomas by using a nomogram model. The purpose of this study was to explore the expression of METTL1 in glioma, its effect on the prognosis of patients, and to elucidate the potential molecular mechanism.

## Methods

### Research design of this study

**Fig. 1:**
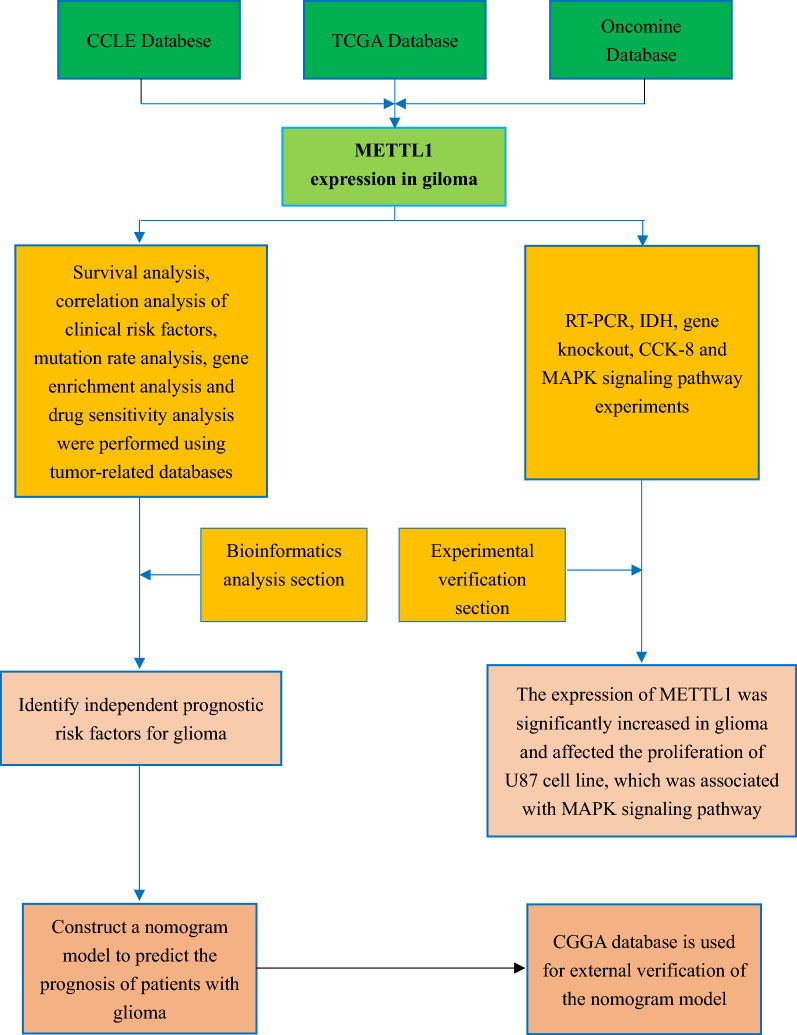
Flow chart of this study

In this study, the expression, prognostic biomarker potential, and molecular mechanism of *METTL1* in glioma were studied and explained using bioinformatics and in vitro experiments (Fig. 1).

### Pan-cancer analysis of METTL1 expression

*METTL1* expression data for normal human tissues and organs were obtained from Genotype-Tissue Expression (GTEx), a public resource containing tissue-specific gene expression and regulation data from multiple normal human tissues and organs. Data of the expression of *METTL1* in different cancer cell lines were obtained from the Cancer Cell Line Encyclopedia database, which accurately displays the genetic characteristics of cancer cells, including 947 human cancer cell lines from more than 30 tissue sources [21]. Additionally, a combination of The Cancer Genome Atlas (TCGA) and GTEx data of METTL1 in pan-cancer were obtained from the UCSC database and RNA sequencing (RNAseq) data was obtained in TPM format after unified processing.

Oncomine is a large tumor gene chip database that contains 86,733 samples from 715 datasets [22] and is mainly used for differential gene expression analysis. Herein, Oncomine was used to identify differentially expressed genes in various cancer tissues with the following query parameters: *p*-value, 0.01; fold change, 1.5; gene rank and data type, all. The obtained data were also used for analyzing the differential expression of *METTL1* across various tumors and between tumor and normal tissues.

### Pan-cancer analysis of METTL1 expression and patient survival

To explore the prognostic value of *METTL1* in various tumors, the relationship between *METTL1* expression and survival of patients with cancer was evaluated. *METTL1* expression data and the survival information of patients, including overall survival (OS) and disease-specific survival (DSS), were obtained from TCGA database. Furthermore, Cox regression and Kaplan–Meier methods were used for survival analysis, and the results were represented as forest maps and survival analysis curves, respectively. Additionally, the relationship between *METTL1* expression and OS and DSS of patients with glioma was evaluated, and a risk score was calculated. The survival of the patients was evaluated by the area under the receiver operating characteristic (ROC) curve (AUC).

### Correlation analysis between METTL1 expression in glioma and clinical risk factors

The clinical information pertaining to the patients with glioma was obtained from TCGA and the Chinese Glioma Genome Atlas (CGGA) databases [23, 24] and was correlated with *METTL1* expression using statistical methods, such as Kruskal − Wallis and Mann − Whitney tests.

### Correlation analysis between METTL1 expression and mutational profile

Tumor mutation burden (TMB) is an important index to evaluate the gene mutation rate. The mutational profile corresponding to the 33 different tumor types was obtained from TCGA database, and the correlation between *METTL1* and TMB in different tumors was represented by a radar map. In addition, the correlation between *METTL1* and TMB in glioma was independently analyzed.

Furthermore, the distribution of *METTL1* mutations, the somatic cell landscape and waterfall maps in glioma, and the distribution of the variants based on mutation classes and single nucleotide variation category were analyzed.

### Gene set enrichment analysis (GSEA)

A total of 697 patients with glioma were selected from TCGA database and divided into high and low expression groups according to the median expression value of *METTL1*. GSEA software was used for enrichment analysis, and the parameters of the comparison group were as follows: high ratio low; several gene set permutations, 1000; datasets, Kyoto Encyclopedia of Genes and Genomes (KEGG) and HALLMARK. The GSEA results having both nominal *p*-value and false discovery rate *q*-value < 0.05 were considered significant.

### Drug target analysis

CellMiner database was used to screen anticancer drugs and their corresponding targets [25]. Based on the comparison of drug and gene target data, different anticancer drugs could be selected for different targets. In this study, data related to gene expression and drug sensitivity score were obtained from the database, and the correlation between *METTL1* expression and drug sensitivity score was evaluated using Student’s *t*-test.

### Real-time reverse transcription-polymerase chain reaction (RT-PCR)

A total of 10 surgically resected glioma tissue samples, including five glioblastoma (GBM), two low-grade gliomas (LGG), and three glioma paracancerous tissues, were evaluated by RT-PCR. The use of primary glioma tissues in this study was approved by the Ethics Committee of the First Affiliated Hospital of China Medical University, and all patients signed informed consent.

Total RNA was extracted using RNAiso Plus reagent (Takara, Kusatsu, Japan; Cat. No. 9108/9109). Reverse transcription and qPCR kits were obtained from ABM (Richmond, BC, Canada; Cat. No. G592, G891, G892) and the experiments were performed according to the manufacturer’s instructions. The thermal cycle parameters for the RT-PCR experiment were as follows: enzyme activation at 95 °C for 3 min; denaturation at 95 °C for 15 s; annealing/extension at 60 °C for 1 min, 40 cycles. The primers for *METTL1* (forward: 5′-AGCTATACCCAGAGTTCTTCGCTCCAC-3′; reverse: 5′-ACAGCCTATGTCTGCAAACTCCACT-3′) and *β-actin* (forward: 5′-AGTGGGGTGGCTTTTAGGATG-3′; reverse: 5′-ACAGCCTATGTCTGCAAACTCCACT-3′) were synthesized by Liuhe BGI Technology Corporation (Beijing, China). The relative expression of *METTL1* determined by RT-PCR was analyzed using the 2^−ΔΔCt^ method.

After conducting the PCR, DNA sequencing was performed to verify the quality of the product. CHROMAS and DNAMAN software and the BLAST function on National Center for Biotechnology Information were used for analysis and similarity comparison with *METTL1* transcripts.

### Immunohistochemistry (IHC)

The specimens used for IHC analysis included tissue samples from 45 patients with glioma and four glioma paracancerous tissues. Among the patients with glioma, 26 were males and 19 were females (age, 16–73 years; mean age, 49.6 years), and a total of 22 and 23 low- and high-grade cases, respectively. Furthermore, 13 patients were alive and 32 had died by the follow-up date. The mean follow-up time was 38.5 months. Survival status and time were collected based on telephonic follow-up. The specimens used for IHC analysis were obtained from the Department of Neurosurgery, First Affiliated Hospital of China Medical University, China.

The primary antibody used was rabbit anti-human polyclonal antibody (Proteintech, Rosemont, IL, USA; Cat. No. 14994-1-AP,). The SP hypersensitive kit was used for secondary antibodies (Fuzhou Maixin Biotech Co., Fuzhou, China). IHC analysis was performed in accordance with the manufacturer’s instructions, and the antibody dilution concentration was determined as 1:100. The stained tissue sections were evaluated by the pathologists and scored according to a semiquantitative scoring method considering the staining intensity and positively stained cell count. Scoring criteria were as follows: (1) no staining (blue color) and positive cell staining ratio < 5%, 0 point; (2) low-intensity staining (yellow color) and positive cell staining between 5 and 25%, 1 point; (3) medium intensity staining (brown color) and positive cell staining between 26%–75%, 2 points; (4) high-intensity staining (brown color) and positive cell staining > 75%, 3 points. Finally, a total score of ≤ 1 was considered as no expression, a score between 2 and 4 was considered as low expression, and a score of > 6 was considered as high expression.

### Cell assay and functional and pathway analysis

Human glioblastoma cell line U87 was purchased from the Chinese Academy of Sciences. Cells were cultured in Dulbecco’s Modified Eagle Medium (DMEM), high glucose (Gibco; No. C11995500bt) containing 10% fetal bovine serum (Bioind; No. 04-001-1A) and 1% antibiotic mixture (Hyclone; No. SV30010) in a humidified incubator with 5% carbon dioxide at 37 °C.

We used siRNA (Ribobio; No.R10043.8) to knock down *METTL1* in the U87 cell line. The transfection efficiency was measured 36 h after transfection using RT-PCR.

The Cell Counting Kit 8 (CCK8) assay was performed using knockdown cells to check cell viability. The U87-knockdown cells (approximately 1,000 cells/well) were inoculated into 96-well plates (6 multiple wells in each group) and the total volume was made up to 100 μl. Thereafter, 10 µl of CCK8 reagent (APExBio; No. K1018) was added to each well 3 h before the target detection time, and the absorbance value (OD) was measured at 450 nm after 3 h of incubation. The data were collected for 4 days, at the same time each day.

Total protein was extracted from U87-knockdown cells using the cell lysate, and placed on a shaker for 15 min for full contact. After centrifugation, the product was obtained, and the protein concentration was determined using the bicinchoninic acid (BCA) protein concentration determination kit (Beyotime; No. P0012s). A fast gel kit (Beyotime; No. P0012AC) was used to prepare the separation and concentrated gels. The concentration of the separation gel was 10%. The protein was transferred to a polyvinylidene fluoride membrane after gel electrophoresis. The membrane was sealed with milk powder and then incubated with primary antibody (GAPDH, ERK1/2 and Phospho-Erk1(T202/Y204) + Erk2(T185/Y187)(p-ERK1/2); Bioss; No. bs-0061R; Abmart; No. T40071, T40072) overnight at 4 ℃, followed by incubation with goat anti-mouse IgG secondary antibody (Bioss; No.bs-40296G-HRP) for luminescence.

### Single-cell RNAseq data analysis

We obtained the raw data of GSE84465 [26] from the web portal and used the "Blueprint Code Data" package for single-cell sequencing analysis. We used the original data to screen more than 6,000 expressed genes, and a total of 14,356 cells were included. After normalization, the characteristics of each sample were analyzed. The most highly dispersed 1,500 genes were selected to construct the homology matrix, and the matrix was then randomly distributed and embedded to obtain a two-dimensional spatial distribution map of all cells.

### Construction and evaluation of the nomogram

The clinical information of patients with glioma from TCGA database along with the high and low expression data of *METTL1* was obtained. First, X-tile software (https://medicine.yale.edu/lab/rimm/research/software/) was used to determine the optimal cutoff value of age, and the following analysis was performed based on the selected results. Next, univariate and multivariate Cox regression models were used to screen the variables, and a *p*-value < 0.05 was considered significant. Finally, a total of five factors were obtained that affected the prognosis of patients with glioma, including *METTL1* expression. These five factors were then used to construct a nomogram, which was evaluated using consistency index (C-index), and ROC, and calibration curves. Additionally, external validation of the nomogram was performed using relevant clinical data from the CGGA database.

### Data processing and statistical analysis

The data analysis and visualizations were performed using R software (version 4.0.1; R Foundation for Statistical Computing, Vienna, Austria). Furthermore, RT-PCR and IHC data were analyzed using Prism 8 (GraphPad Software, San Diego, CA, USA). A *p*-value < 0.05 was considered statistically significant.

## Results

### METTL1 expression across cancer and normal tissues

**Fig. 2:**
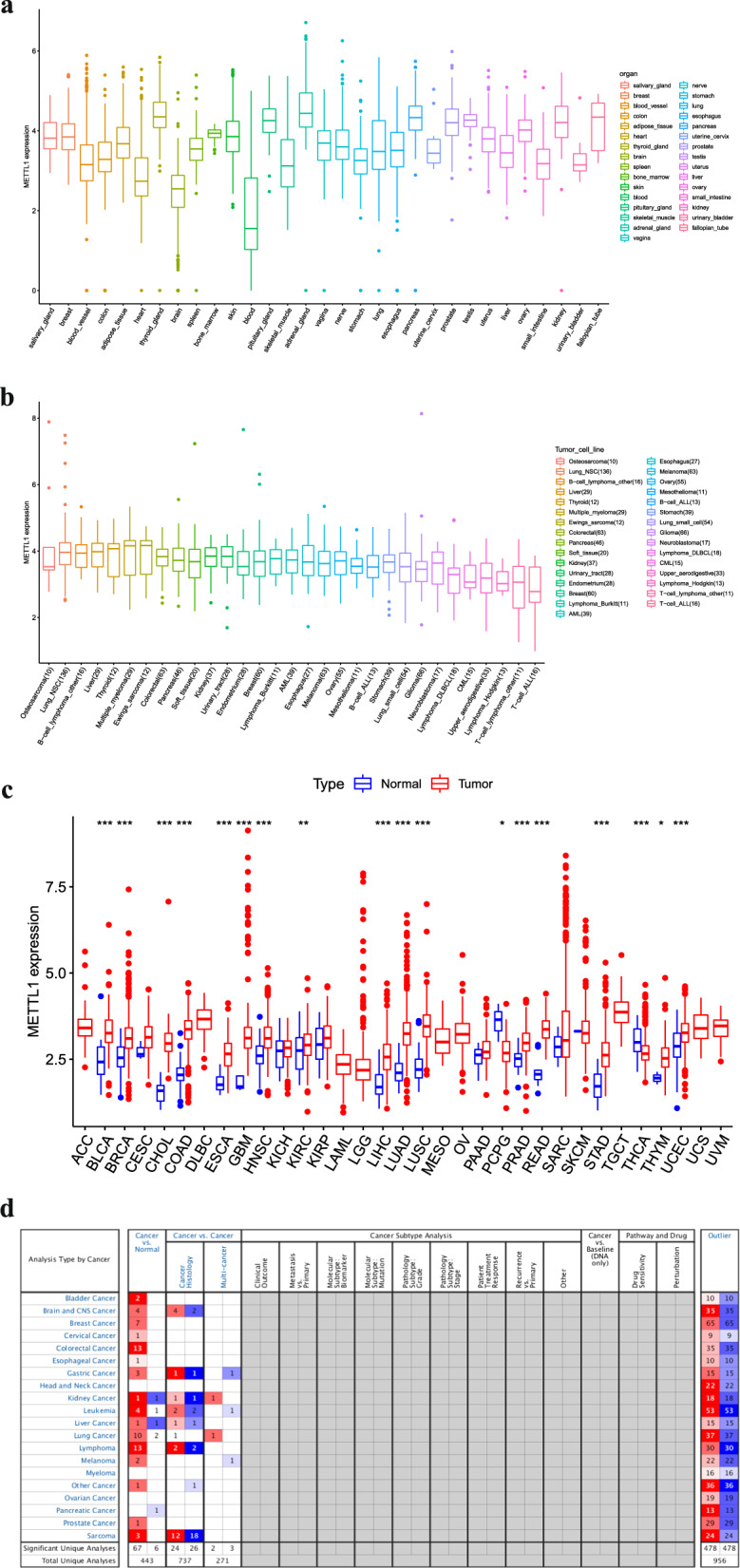
Expression of METTL1 in different normal tissues and tumors.** a** METTL1 expression in 31 normal human tissues. **b** METTL1 expression in 31 tumor cell lines.** c** Pan-cancer analysis of METTL1 expression in TCGA database. (*: P < 0.05; **: P < 0.01; ***: P < 0.001). **d** Pan-cancer analysis of METTL1 expression in Oncomine database

The expression of *METTL1* was found to be lower in normal human blood and brain tissues than in other normal tissues (Fig. 2a), whereas it was similar across various cancer cell lines (Fig. 2b). Nevertheless, *METTL1* expression was found to be significantly higher in bladder urothelial carcinoma, breast invasive carcinoma, cholangiocarcinoma, colon adenocarcinoma, esophageal carcinoma, GBM, head and neck squamous cell carcinoma, kidney renal clear cell carcinoma (KIRC), liver hepatocellular carcinoma (LIHC), lung adenocarcinoma, lung squamous cell carcinoma, prostate adenocarcinoma, rectum adenocarcinoma, stomach adenocarcinoma, thyroid carcinoma, thymoma, and uterine corpus endometrial carcinoma tissues than in the corresponding normal tissues (Fig. 2c). In contrast, *METTL1* expression was found to be significantly lower in pheochromocytoma and paraganglioma (PCPG) than in the corresponding normal tissue (Fig. 2c). Furthermore, data from Oncomine databases showed that METTL1 expression varied significantly across tumor tissues. The tumors that showed significant differences included colorectal cancer and leukemia (Fig. 2d).

### Correlation between METTL1 expression and survival of patients with cancer

**Fig. 3:**
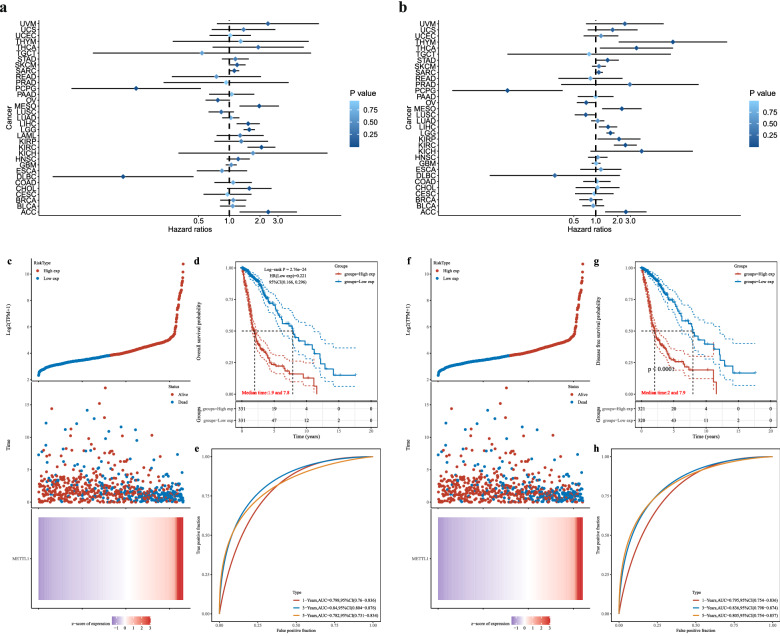
Pan-cancer analysis of METTL1 expression and patient survival. **a**, **b** Forest map of the relationship between METTL1 expression and prognosis in 33 tumors, OS and DSS independently. **c**–**e** METTL1 in OS, risk score and heat map, KM survival analysis curve and ROC curve. **f**–**h** METTL1 in DSS, risk score and heat map, KM survival analysis curve and ROC curve

Survival analysis was performed by comparing the relationship between *METTL1* expression and survival time and status of the patients with cancer. The results obtained using Cox regression analyses are represented as forest maps (Fig. 3a, b), which showed a significant relationship between *METTL1* expression and prognosis and survival of patients with adrenocortical carcinoma, KIRC, LGG, LIHC, and mesothelioma. Furthermore, the survival curve analysis indicated a significant relationship between the expression of *METTL1* and OS and DSS of patients with KIRC, LGG, LIHC, mesothelioma, ovarian serous cystadenocarcinoma, and PCPG.

In the case of patients with glioma, p values in survival analysis were all less than 0.001, showing significant statistical differences (Fig. 3d, g). Survival analysis showed that high expression of *METTL1* correlated with a higher risk score and that most patients who died were in the high-risk group (Fig. 3c, f). In addition, the AUC values for 1-, 3-, and 5-year survival were 0.798, 0.84, and 0.782, respectively (Fig. 3e, h), indicating the reliability of the survival analysis.

**Fig. 4:**
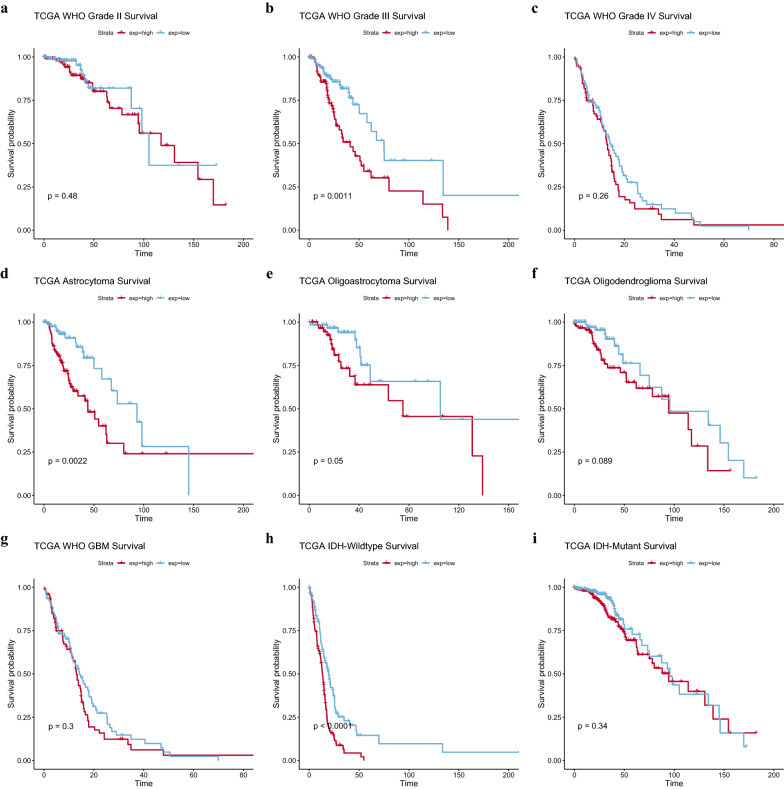
Survival analysis of different types of gliomas. **a**–**c** Survival curves for gliomas of different grades. **d**–**g** Survival curves for gliomas of different histological type. **h**–**i** Survival curves for gliomas of different IDH mutation status

Among different World Health Organization (WHO) grades, histological types, and isocitrate dehydrogenase (IDH) mutation statuses, the survival of patients with WHO III astrocytoma and IDH wild type was statistically significant (Fig. 4b, d, h).

### Correlation between METTL1 expression and clinical risk factors

**Fig. 5:**
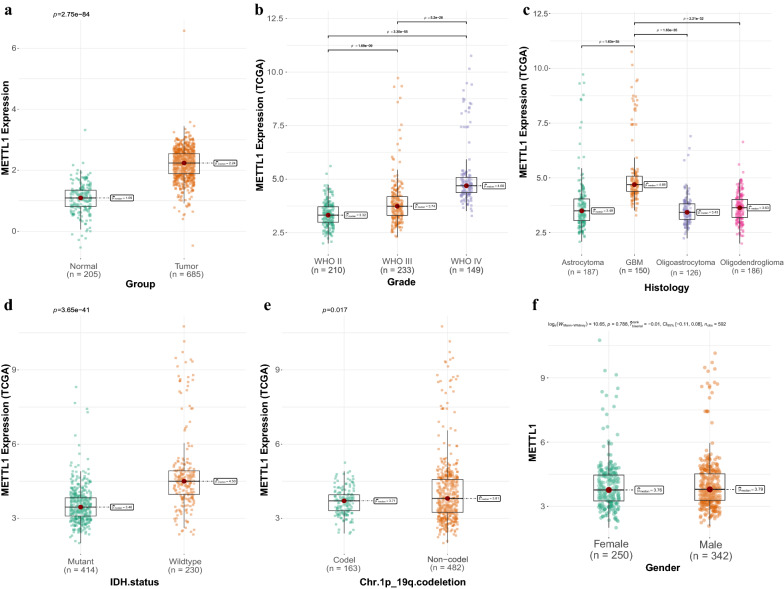
The relationship between METTL1 expression and clinical risk factors in TCGA and CGGA database

**Fig. 6:**
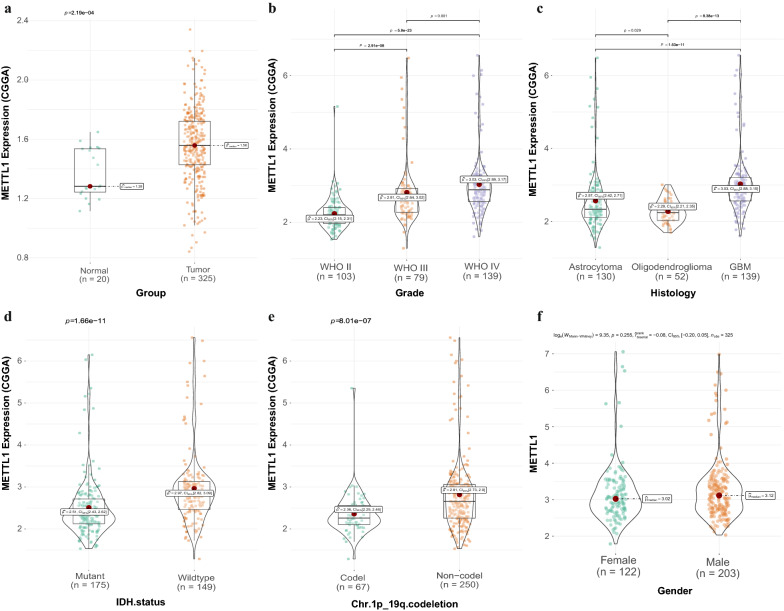
The relationship between METTL1 expression and clinical risk factors in TCGA and CGGA database

Correlation analysis indicated that *METTL1* expression correlated with the WHO classification (p values were all less than 0.001), histological type (p values were all less than 0.05), IDH mutation status (p_TCGA_ = 3.65e−41; p_CGGA_ = 1.66e−11), and 1p19q codeletion (p_TCGA_ = 0.017; p_CGGA_ = 8.01e−07) based on data obtained from both TCGA (Fig. 5a–e) and CGGA (Fig. 6a–e) databases.

### METTL1 mutational status across cancer types

**Fig. 7:**
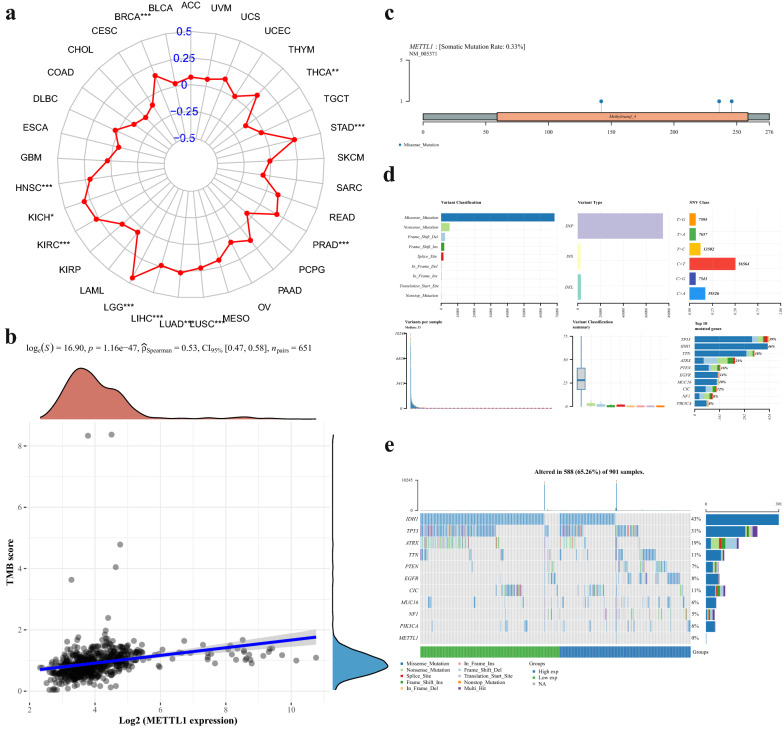
The relationship between METTL1 expression and mutation. **a** METTL1 expression in 33 tumors was correlated with TMB.** b** PRMT6 expression in glioma was correlated with TMB score. **c** Lollipop map of mutation distribution of METTL1 in glioma. **d** Summary glioma plot displays the distribution of variants by variant classification, type, and SNV category.The bar chart shows the top ten mutated genes. **e** Oncoplot shows the landscape of somatic mutations in gliomas, sorted by genetic mutation

A significant correlation between *METTL1* expression and TMB was observed in multiple tumor types, specifically in LGG (Fig. 7a). However, the correlation score in all tumor types was < 0.5, whereas in glioma samples, it was found to be 0.53 (Fig. 7b). Hence, a low correlation was found between *METTL1* expression and mutation in glioma samples and the mutation rate was only 0.33%. In addition, the analysis of somatic mutation rates, mutation information in each sample, and variation types (Fig. 7c–e) indicated that the mutation rate of *METTL1* was low in patients with glioma. In addition, the mutation of METTL1 in 901 samples in TCGA database was almost zero.

### Identification of pathways and potential drug candidates for METTL1

**Fig. 8:**
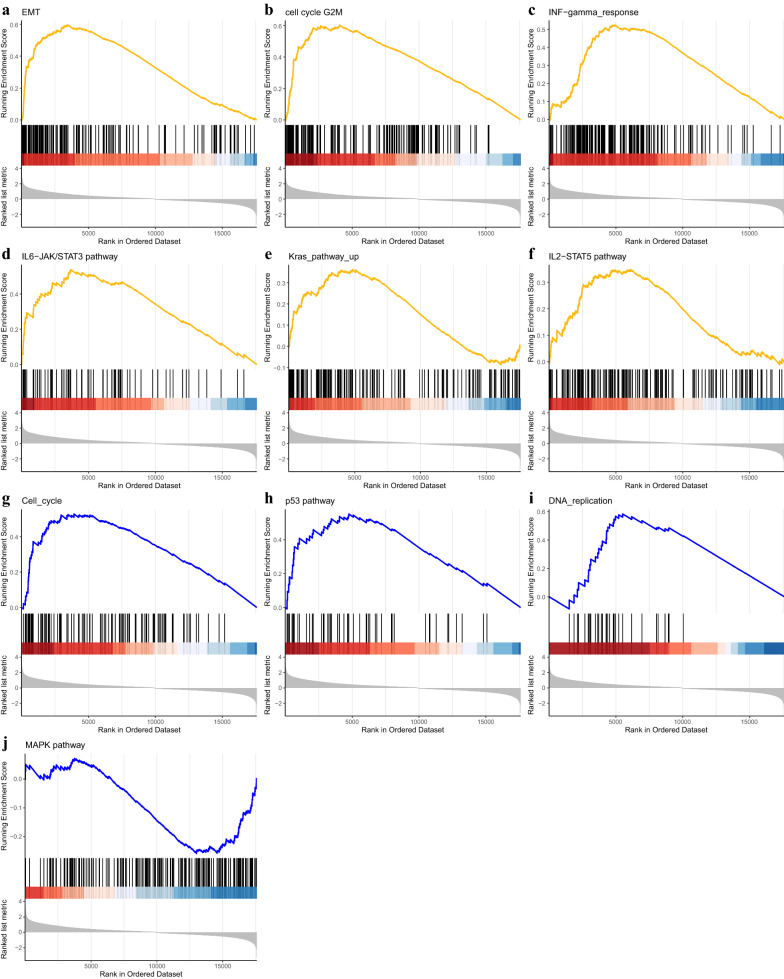
Results of gene enrichment analysis. a. Gene enrichment KEGG analysis. **a**–**f** Gene enrichment HALLMARK analysis on the enrichment results of METTL1. **g**–**j** Gene enrichment KEGG analysis on the enrichment results of METTL1

To explore the potential molecular mechanism of *METTL1* in glioma, pathway and functional enrichment analyses were performed based on the expression of *METTL1*. As shown in Fig. 8, functions, such as cell cycle, DNA replication, and epithelial-mesenchymal transition, were enriched, while pathways, such as p53, MAPK, IL6-JAK-STAT3, and IL2-STAT5 signaling were enriched (Table 1).

**Table 1:** Gene set enrichment analysis of METTL1

Gene set names	NOM p-val	FDR q-val
KEGG gene set		
KEGG_CELL_CYCLE	1.61E−08	0.000000465
KEGG_P53_SIGNALING_PATHWAY	0.000027	0.000348425
KEGG_DNA_REPLICATION	0.0001745	0.001727684
KEGG_MAPK_SIGNALING_PATHWAY	0.0035289	0.020070548
HALLMARK gene set		
HALLMARK_EPITHELIAL_MESENCHYMAL_TRANSITION	1E−10	4.05E−09
HALLMARK_G2M_CHECKPOINT	1E−10	4.05E−09
HALLMARK_INTERFERON_GAMMA_RESPONSE	1E−10	4.05E−09
HALLMARK_IL6_JAK_STAT3_SIGNALING	0.0000015	0.000028
HALLMARK_KRAS_SIGNALING_UP	0.0031269	0.018333216
HALLMARK_IL2_STAT5_SIGNALING	0.0063964	0.031755704

Gene sets with NOM p-val and FDR q-value < 0.05 are considered as significant

**Fig. 9:**
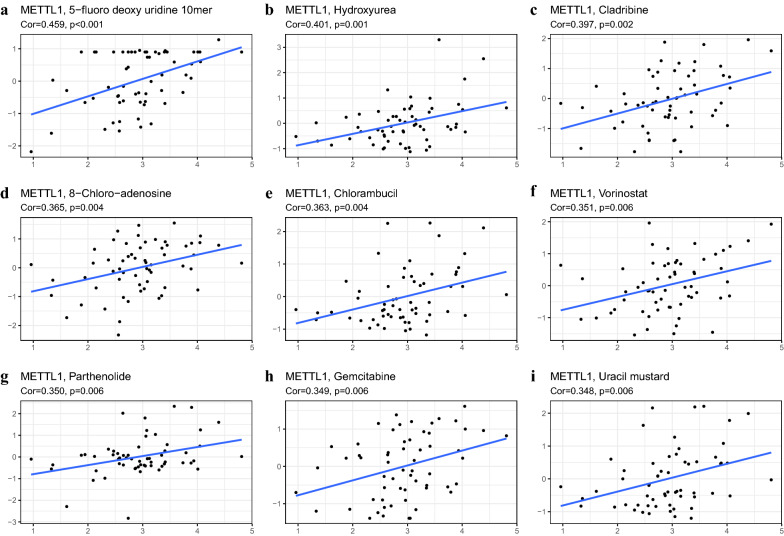
Drug target analysis. **a**–**i** METTL1 and chemotherapeutic agents were analyzed for sensitivity, and ranked by p-value and correlation

Furthermore, the correlation score between two antitumor drugs 5-fluoro-deoxy-uridine and hydroxyurea, and *METTL1* expression was found to be 0.459 and 0.401, respectively, with p values less than or equal to 0.001 (Fig. 9).

### Experimental validation of METTL1 expression in glioma

Total RNA from primary glioma samples was analyzed by spectrophotometry and the OD260/OD280 ratio was found to be between 1.9 and 2.1, which indicated good purity, and the RNA was used for subsequent experiments. The solution curve of *METTL1* and *β-actin* was unimodal, whereas the amplification curve was S-shaped, indicating that the reaction system had no specific fluorescence, and thus the quantitative results were reliable (Additional file 2a, b). Furthermore, *METTL1* expression was found to be significantly higher in glioma than in the adjacent normal tissues (Additional file 2c). In addition, results of DNA sequencing performed to verify the accuracy of the PCR products showed that the product and target gene transcriptome similarity was more than 98.5% (Additional file 2).

**Fig. 10:**
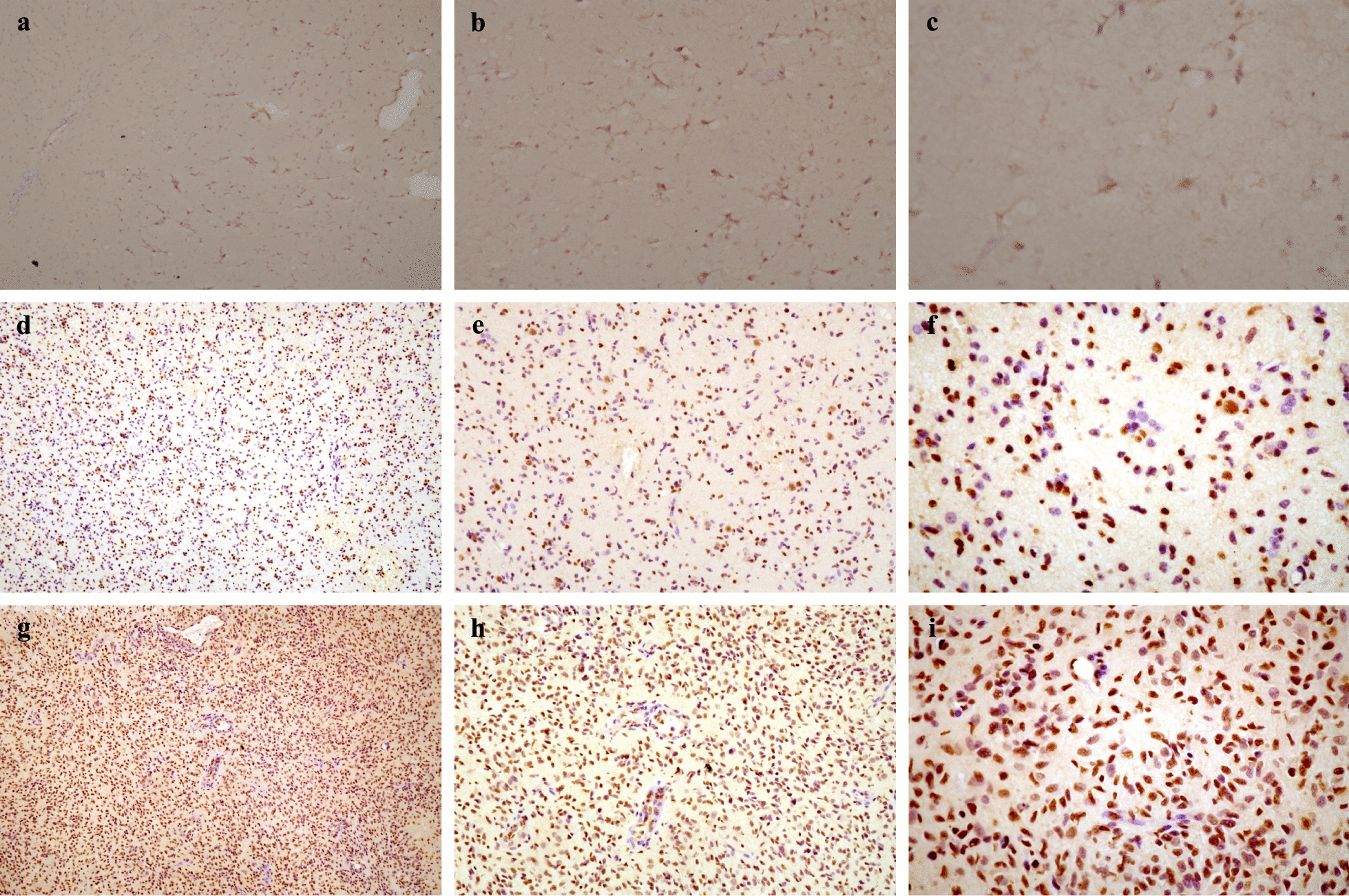
Microscopic image of IDH. **a**–**c** IHC staining of paracancerous tissues with METTL1 were 100-, 200- and 400- fold microscopic images. **d**–**f** IHC staining of LGG with METTL1 were 100-, 200- and 400- fold microscopic images. **g**–**i** IHC staining of GBM with METTL1 were 100-, 200- and 400- fold microscopic images

**Fig. 11:**
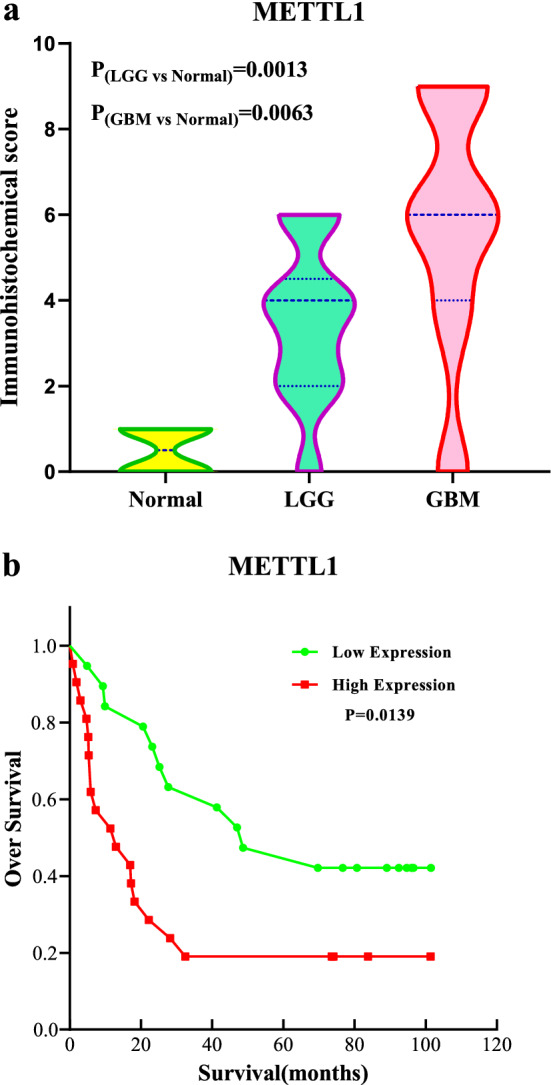
Immunohistochemical results.** a** Violin plot of METTL1 IHC scores in glioma and paracancerous tissues. **b** Survival analysis of high and low expression of METTL1 in IHC score

IHC analysis of glioma samples indicated that *METTL1* was localized in the nucleus. Furthermore, *METTL1* levels were found to be high in 21 cases and low in 19 cases, whereas it was not detected in 5 cases. The microscopic histochemical images of paracancerous tissue, LGG, and GBM at a magnification of 100, 200, and 400× , respectively, are shown in Fig. 10. The results indicated that the expression of METTL1 significantly increased with tumor grade, having the highest expression in GBM followed by that in LGG and adjacent tissues (Fig. 11a). In addition, the patients with high METTL1 expression had a worse prognosis and shorter survival time (Fig. 11b).

### Verification of the role of METTL1 in glioma in vitro

**Fig. 12:**
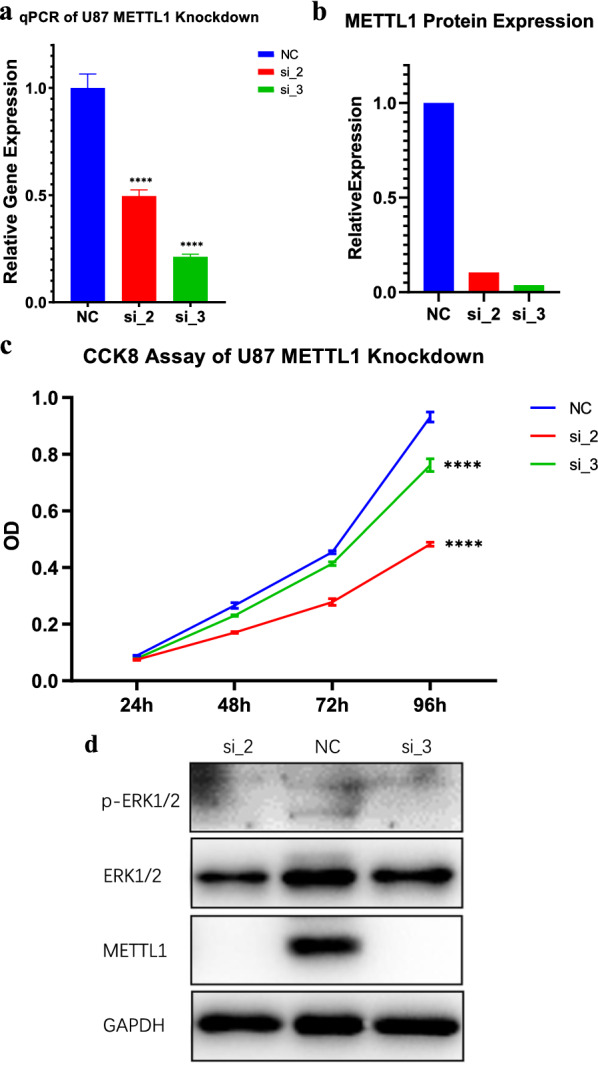
Cell proliferation assay and pathway analysis. **a**, **b** Effects of METTL1 knockdown at the RNA and protein levels. **c** Results of cell proliferation experiment. **d** Pathway experiment results

In the knockdown experiment, we used three sequences and selected two of them for demonstration. First, a PCR experiment was conducted to verify the knockdown efficiency of *METTL1*. As can be seen from Fig. 12a, the knockdown rate reached 70% at the RNA level, while the protein level was also significantly reduced and the knockout rate of protein level was more than 80% (Fig. 12b). As can be seen from the Fig. 12c, the cell proliferation level of METTL1 knockdown was significantly lower than that of wild type cells at 48 h, and the proliferation difference increased gradually with time. Representative targets in the mitogen-activated protein kinase (MAPK) pathway were selected for verification. Figure 12d shows that the knockdown of *METTL1* has an inhibitory effect on p-EPK1/2.

### Single-cell sequencing results of METTL1

We processed the raw data through the R package "Seurat pipeline" and divided all cells into 13 clusters (Additional file 3a). Furthermore, we annotated these populations into five cell types, namely adipocytes, astrocytes, fibroblasts, macrophages, and neurons (Additional file 3b). The expression of *METTL1* tended to increase in clusters 1, 2, and 3 and especially in cluster 6 (Additional file 3c).

### Correlation between METTL1 expression and clinical risk factors

**Fig. 13:**
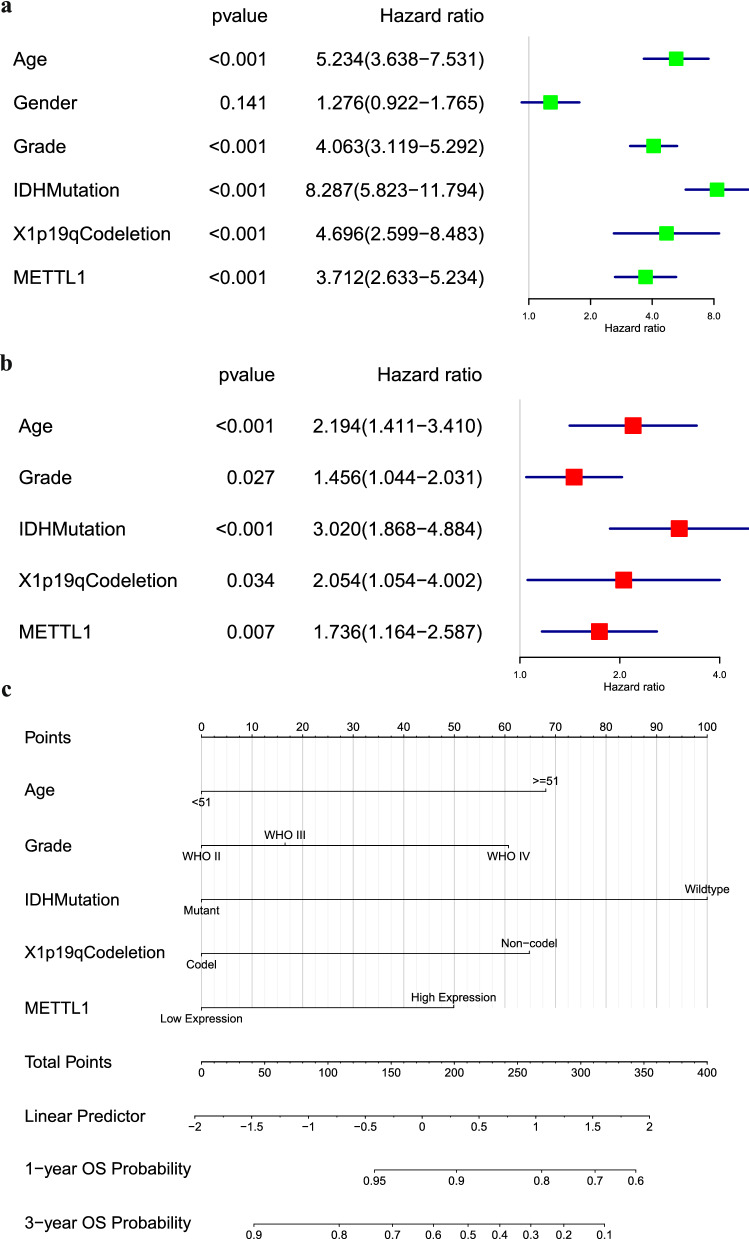
Screening of independent risk factors and construction of the nomogram. **a** Univariate Cox regression analysis results based on CGGA database. **b** Multivariate Cox regression analysis results based on CGGA database. **c** Nomogram based on CGGA data. **d** Calibration curve of nomogram based on TCGA data. **e** Calibration curve of nomogram based on CGGA data

**Fig. 14:**
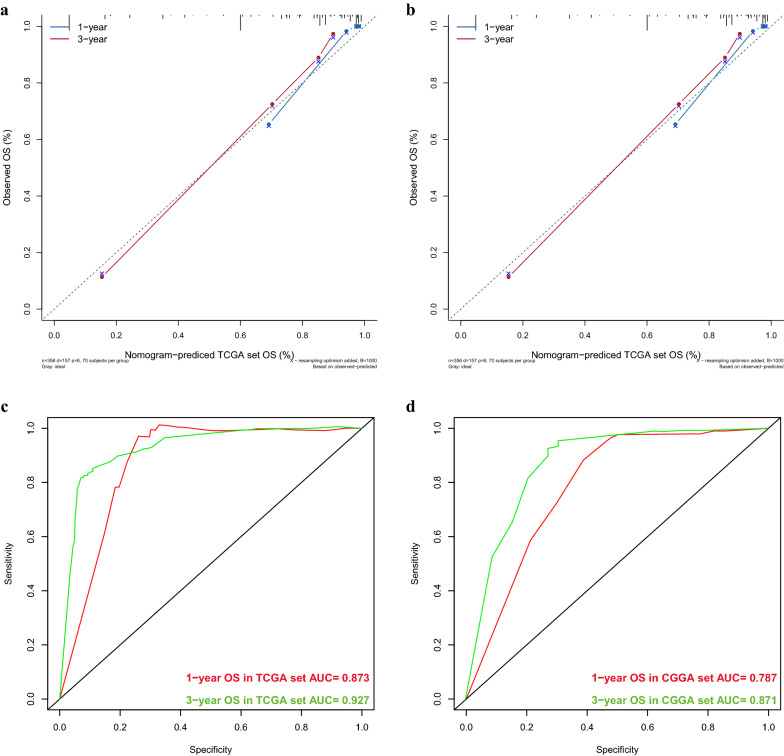
Evaluation and external validation of the nomogram.** a** Calibration curve of nomogram based on CGGA data. **b** Calibration curve of nomogram based on TCGA data. **c** The ROC curve of the nomogram built based on CGGA data. **d** The ROC curve of the nomogram built based on TCGA data

Next, various clinical risk factors were evaluated using the Cox regression model. Age (p < 0.001, p < 0.001), WHO grade (p < 0.001, p = 0.027), IDH mutational status (p < 0.001, p < 0.001), 1p19q codeletion status (p < 0.001, p = 0.034), and the expression (high or low) of *METTL1* (p < 0.001, p = 0.007) (Fig. 13a, b) were the selected risk factors used to construct a nomogram and predict 1- and 3-year survival rates of patients with glioma (Fig. 13c) 0.16 The nomogram results indicated that the C-index of the training and validation sets were 0.841 (95% confidence interval: 0.817–0.865) and 0.765 (0.734–0.796), respectively. Furthermore, the training and validation sets indicated that the calibration curves for 1- and 3-year survival rates were predicted to be close to the standard curves (Fig. 14a, b). The AUC values in the training and validation sets for the 1-year survival rate were 0.873 and 0787, respectively (Fig. 14c), whereas those for the 3-year survival rate were 0.927 and 0.871, respectively (Fig. 14d). These results show that the constructed nomogram was highly reliable, demonstrating good efficiency in external validation.

## Discussion

RNA methylation plays an important role in the progression of cancer and has been extensively studied in recent years. The transformation of normal cells into cancerous cells requires the gain-of-function of oncogenes, which result in mutations, or the loss-of-function of tumor suppressor genes [27]. The RNA methyltransferase enzyme plays an important role in promoting the transformation of normal cells to cancer cells [28]. *METTL1* catalyzes the formation of 7-methylguanosine (m^7^G), mainly at position 46 of the transfer RNA sequence. In addition, it similarly affects mRNA and regulates its translation [5]. In this study, the differential expression and prognostic importance of *METTL1* were evaluated in various cancer types using multiple bioinformatics databases. The collected data demonstrated that *METTL1* is potentially important in various tumors, especially in glioma. Furthermore, the expression of *METTL1* in different grades of glioma tissues was evaluated by RT-PCR and IHC, which revealed that *METTL1* expression was significantly different between normal brain and glioma tissues, and increased with increasing grades of glioma. Moreover, *METTL1* expression is closely associated with common clinical risk factors, such as IDH mutational status and 1p19q codeletion status, among others. Notably, *METTL1* expression is generally elevated in patients with IDH wild-type glioma, and IDH mutation is believed to play an important role in early glioma development [29, 30]. Results of the in vitro experiments revealed that METTL1 affects the proliferation of glioma. Based on this evidence, it is reasonable to hypothesize that *METTL1* expression is intrinsically associated with the risk factors significantly associated with glioma prognosis. In addition, this study obtained relevant survival time and clinical information through follow-up of patients with glioma in our hospital. Through survival analysis, we found that patients with high METTL1 expression had a shorter survival time and poor prognosis. Thus, based on the above findings, we noted the important prognostic value of METTL1 in patients with glioma, indicating that METTL1 is a potential tumor marker.

Due to the limited treatment options for glioma, targeted therapeutic strategies are gaining attention in recent times and are being applied in clinical settings. Few studies have reported different therapeutic targets, such as mTOR, EGFR, and VEGFR, for the treatment of glioma [31–33]. However, there have been very few studies reporting m^7^G methylation sites as therapeutic targets, especially in glioma. The present study showed that increased expression of *METTL1* is significantly correlated with the prognosis of patients and different clinical risk factors, suggesting that *METTL1* may serve as a promising therapeutic target for glioma. In addition, according to the analysis results of antitumor drugs, 5-fluoro-deoxy-uridine and hydroxyurea have a strong correlation with METTL1, and studies have shown that combination chemotherapy with hydroxyurea and other drugs has a certain therapeutic effect on malignant glioma [34, 35]. Furthermore, the nomogram results suggested that *METTL1* may serve as an important potential prognostic risk factor for glioma.

Additionally, based on bioinformatic analyses, the probability of occurrence of mutations in *METTL1* in glioma was very low; hence, *METTL1*-mediated m^7^G methylation may be important for the development of glioma. The methylation of RNA affects the synthesis of proteins in various ways, including the accuracy of translation [36, 37] and the overall structure of the modified RNA [38]. Furthermore, RNA methylation affects the stability of the RNA itself, thus affecting the abundance of RNA molecules in cells [39, 40]. Moreover, the methylation process leads to RNA polymerase III-dependent transcriptional regulation that promotes the abundance of intracellular transfer RNA types, which has been demonstrated to be an effect of oncogenic signaling pathways [41]. Cancer cells are known to have a higher overall rate of protein synthesis than their normal counterparts, which in turn increases their ability to proliferate [42, 43]. Furthermore, m^7^G methylation has been known to increase translation efficiency and microRNA processing [44, 45]. Additionally, it has been suggested that *METTL1* plays a key role in maintaining stem cell stability through p53/WNT/FGF signaling [46, 47]. Moreover, knocking down the expression of *METTL1* has been shown to alter the self-renewal ability of stem cells [48, 49] as well as the cell circulation process. The findings of this study show that METTL1 also affects the proliferation process of gliomas. The results of our study are similar to those of other studies that have shown that METTL1 also promotes tumor proliferation in liver and colon cancers [50, 51]. In addition, the interaction of *METTL1* with the p53 signaling pathway in other tumors has been evaluated and described previously [52, 53], and overexpression of *METTL1* has been the common denominator in all these studies. In this study, the MAPK signaling pathway was explored; according to the results, *METLL1* knockdown affects the core targets of this pathway to a certain extent.

The present study has some limitations. Only a small number of glioma samples were analyzed using RT-PCR. A relatively small number of targets have been identified in the MAPK pathway, and it is necessary to further study the relationship between METTL1 and this pathway.

## Conclusions

This study is the first to explore the prognostic value of METTL1 in gliomas and it was found that the expression of *METTL1* increases with the grade of glioma. Additionally, it affects glioma proliferation and has a potential association with MAPK signaling pathway. However, the biological behavior, potential mechanism and pathway of METTL1 on glioma need to be further studied. Furthermore, based on the results of the nomogram, the expression of *METTL1* may be used as a potential independent prognostic risk factor. Finally, using publicly available databases and collected clinical data, *METTL1* was shown to be a potential biomarker for glioma that serves as a promising therapeutic target.

## Supplementary Information

**Additional file 1.** Results of RT-PCR. (a). The amplification curve of METTL1 and β-actin. (b). The solution curve of METTL1 and β-actin. (c). A histogram of METTL1 RT-PCR results in glioma and paracancerous tissues.

**Additional file 2.** DNA sequencing results from all the samples.

**Additional file 3.** (a). Distribution of 13 clusters. (b). The cells of each cluster were classified. (c). Expression and distribution of METTL1 in each cluster.

**Additional file 4**. (a). The uncropped western strip with METTL1 knocked down. (b). The uncropped western strip with EPK. (c). The uncropped western strip with p-EPK.

## Data Availability

The data in the bioinformatics part of this study were all from open databases.

## Abbreviations

AUC: Area under the ROC
CGGA: Chinese Glioma Genome Atlas
DSS: Disease-specific survival
GSEA: Gene set enrichment analysis
GMB: Glioblastoma
IDH: Isocitrate dehydrogenase
IHC: Immunohistochemistry
KIRC: Kidney renal clear cell carcinoma
LIHC: Liver hepatocellular carcinoma
LGG: Low-grade glioma
m^7^G: N(7)-methylguanine
mRNA: Messenger RNA
METTL1: Methyltransferase-like 1
OS: Overall survival
PCPG: Pheochromocytoma and paraganglioma
ROC: Receiver operating characteristic
RT-PCR: Reverse transcription-polymerase chain reaction
TMB: Tumor mutation burden
WHO: World Health Organization
