# Supplementary figures and images for: Prognostic role of METTL1 in glioma
Source: Cancer Cell Int. 2021 Nov 27;21:633. doi: 10.1186/s12935-021-02346-4 (PMC8627054; doi:10.1186/s12935-021-02346-4)

**a**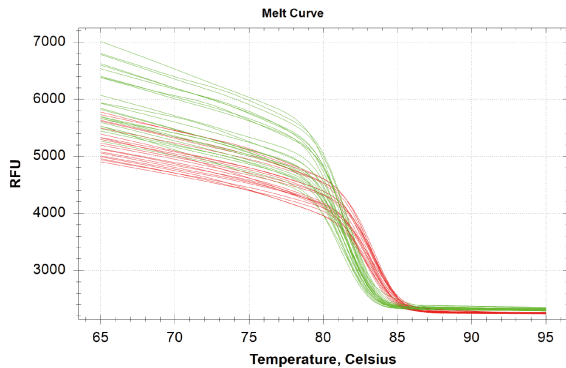**b**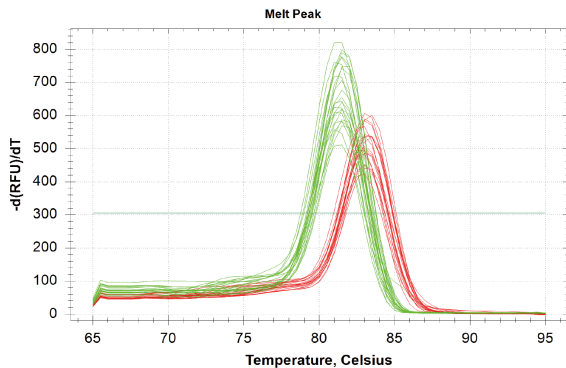**c**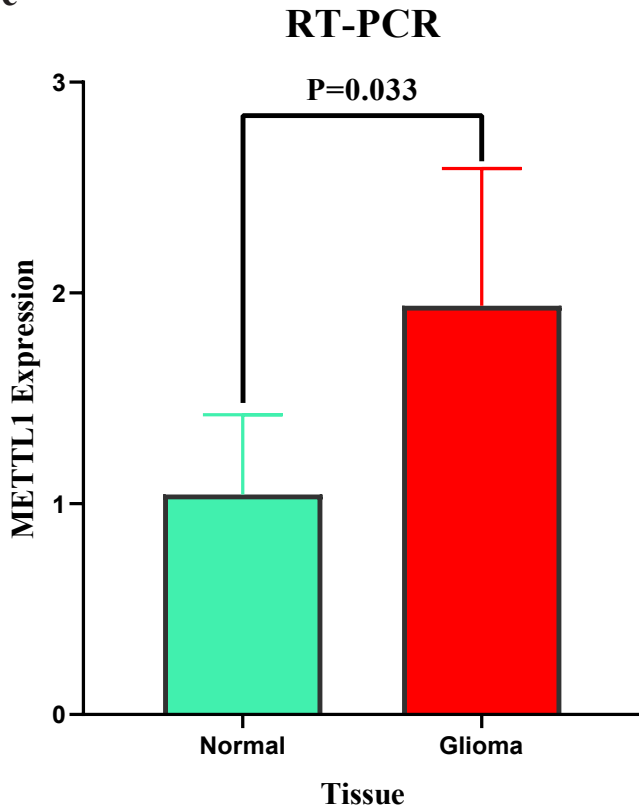

Supplement: Supplementary file 1 — Additional file 1. Results of RT-PCR. (a). The amplification curve of METTL1 and β-actin. (b). The solution curve of METTL1 and β-actin. (c). A histogram of METTL1 RT-PCR results in glioma and paracancerous tissues. [file 12935_2021_2346_MOESM1_ESM.pdf]

**a**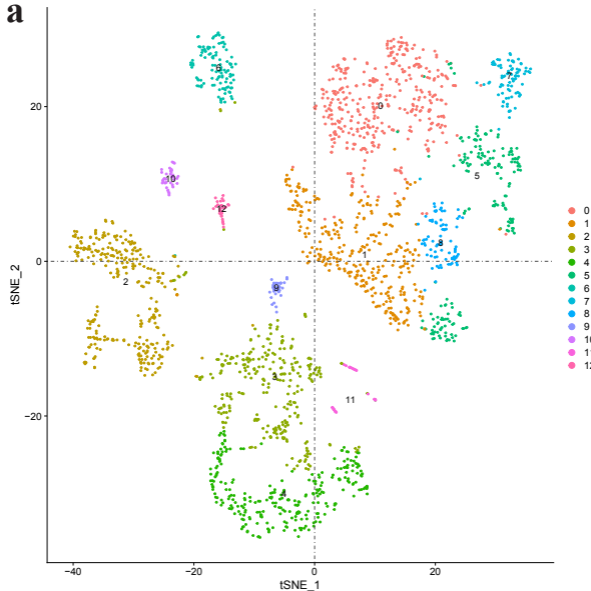**b**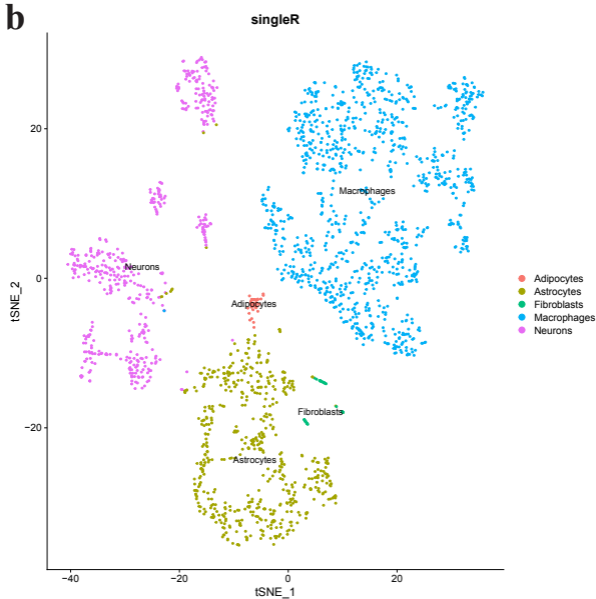**c**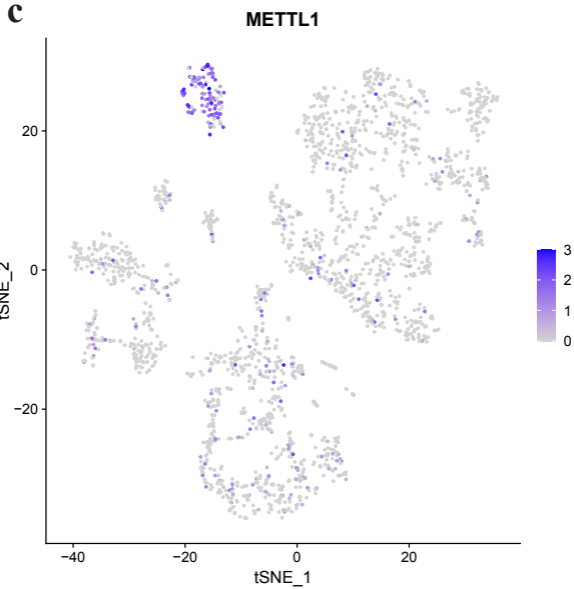

Supplement: Supplementary file 3 — Additional file 3. (a). Distribution of 13 clusters. (b). The cells of each cluster were classified. (c). Expression and distribution of METTL1 in each cluster. [file 12935_2021_2346_MOESM3_ESM.pdf]

**a**

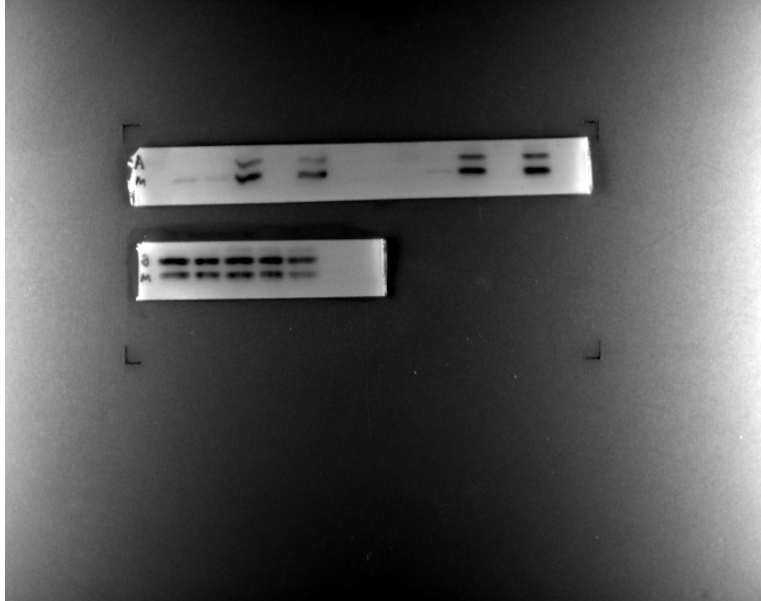

**b**

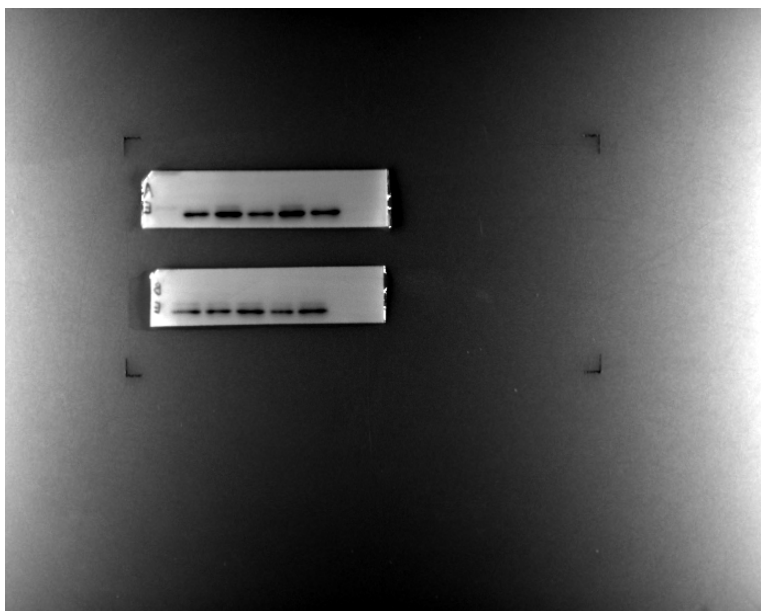

**c**

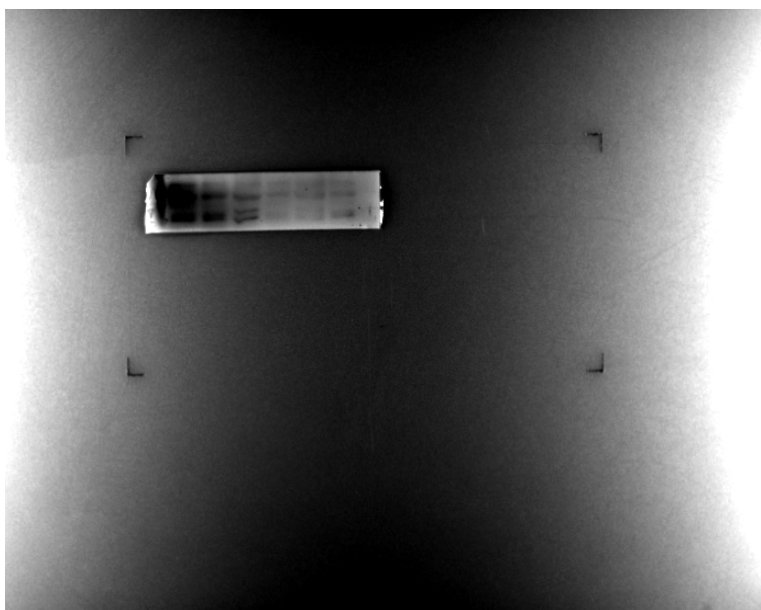

Supplement: Supplementary file 4 — Additional file 4. (a). The uncropped western strip with METTL1 knocked down. (b). The uncropped western strip with EPK. (c). The uncropped western strip with p-EPK. [file 12935_2021_2346_MOESM4_ESM.pdf]
